# Hybrid Microfluidic Platform for Multifactorial Analysis Based on Electrical Impedance, Refractometry, Optical Absorption and Fluorescence

**DOI:** 10.3390/mi7100181

**Published:** 2016-10-07

**Authors:** Fábio M. Pereira, Iwona Bernacka-Wojcik, Rita S. Rodrigues Ribeiro, Maria Teresa Lobato, Elvira Fortunato, Rodrigo Martins, Rui Igreja, Pedro A. S. Jorge, Hugo Águas, Abel Martin Gonzalez Oliva

**Affiliations:** 1 CENIMAT/I3N, Department of Materials Science, Faculty of Science and Technology, New University of Lisbon and CEMOP/UNINOVA, 2829-516 Caparica, Portugal; fmrp89@hotmail.com (F.M.P.); iwona.bernacka.wojcik@liu.se (I.B.-W.); mt.almeida@campus.fct.unl.pt (M.T.L.); emf@fct.unl.pt (E.F.); rm@uninova.pt (R.M.); rni@fct.unl.pt (R.I.); 2 Instituto de Tecnologia Química e Biológica Antonio Xavier, Universidade Nova de Lisboa, Avenida da Republica, 2780-157 Oeiras, Portugal; 3 Faculdade de Ciências, Universidade do Porto, Rua do Campo Alegre, 687, 4169-007 Porto, Portugal; ritbeiro@gmail.com; 4 Centre for Applied Photonics, Institute for Systems and Computer Engineering Technology and Science (INESC TEC), Rua do Campo Alegre, 687, 4150-179 Porto, Portugal; pedro.jorge@fc.up.pt

**Keywords:** single cell analysis, label-free methods, impedance spectroscopy, refractometry, optical absorption, hybrid microfluidic chip

## Abstract

This paper describes the development of a novel microfluidic platform for multifactorial analysis integrating four label-free detection methods: electrical impedance, refractometry, optical absorption and fluorescence. We present the rationale for the design and the details of the microfabrication of this multifactorial hybrid microfluidic chip. The structure of the platform consists of a three-dimensionally patterned polydimethylsiloxane top part attached to a bottom SU-8 epoxy-based negative photoresist part, where microelectrodes and optical fibers are incorporated to enable impedance and optical analysis. As a proof of concept, the chip functions have been tested and explored, enabling a diversity of applications: (i) impedance-based identification of the size of micro beads, as well as counting and distinguishing of erythrocytes by their volume or membrane properties; (ii) simultaneous determination of the refractive index and optical absorption properties of solutions; and (iii) fluorescence-based bead counting.

## 1. Introduction

Miniaturization and portability, increased automation, minimum reagent consumption, high throughput and reduced manufacturing costs are some of the strongest motivations towards the development of microfluidic platforms [1,2]. However, scaling down very often imposes limitations on the usable detection methods. In this regard, the very high sensitivity and intrinsic scalability of some analytical optical methods make them appropriate choices for incorporation in microfluidic sensing platforms [3]. Indeed, optical techniques based on absorption/fluorescence spectroscopy [4] or refractive index-based label-free detection schemes [5] are among the most popular approaches for lab-on-a-chip chemical and biological sensing applications [6]. On the other hand, a diversity of systems taking advantage of electrical-based methods such as electrochemical sensing, impedance analysis or capillary electrophoresis, have been reported [7].

While a large number of successful devices and applications have been reported using either one of these techniques, very little was achieved on the combined usage of different detection methods in the same platform. However, it is recognized that the simultaneous use of multiple techniques to characterize the same sample can provide more complete information and allow better discrimination in a diversity of diagnostic and analytical applications. This is particularly true, for instance, in environmental applications where the ability to handle complex and highly variable sample matrices in microfluidic devices is of paramount importance [2]. 

In this context, efforts are underway to provide multifactorial analysis in a single chip, using a combination of analytical techniques, to address complex samples. For instances, recently, dual technique measurements were performed where the refractive index and absorption spectra of the dye Rhodamine 6G in glycerol were investigated using a single microfluidic system [8]. The refractive index was measured by shining a collimated laser diode (635 nm) into the microfluidic channel and monitoring the interference pattern of the scattered light with a charged-coupled device (CCD) camera. On the other hand, a white light-emitting diode (LED) and a fiber optic spectrometer were used to acquire the sample absorption spectra. All of the optic signals were coupled in and out of the chip using bulk optics. In a more integrated approach, Barat et al. proposed a hybrid measurement using on-chip electrodes and optical fibers to simultaneously acquire the impedance and fluorescence spectra of polystyrene beads [9].

Perhaps some of the most representative applications benefiting from a multifactorial analysis are in the field of single cell analysis and diagnostics. For instance, the sensitivity of impedance-based analysis alone can be limited by specific cell properties that make their identification less accurate, such as cell age, shape, size, chemical composition, infection stage, media conductivity, matrix complexity, etc. [10]. In principle all of these characteristics can be detected; however, owing to system complexity, it is difficult to extract information from the data, as the results are the addition of several overlapping contributions [11,12,13]. In this context, simultaneous analysis by various sensing methods can provide a larger amount of data for each biologic subject analyzed, allowing the correlation of the multidimensional information towards a better characterization of the target.

To complement the information acquired by impedance spectroscopy, optical measurements are particularly suited, as they can be straightforwardly implemented in microfluidic devices. Recently, various groups have developed microfluidic cytometers that enable simultaneous single cell analysis by impedance and fluorescence-based methods [9,12,13,14]. Cell autofluorescence could be used for label-free detection of pathogens, as different stages of pathologies yield alterations in endofluorophores activity affecting the autofluorescence spectral shape [15]. On the other hand, absorption spectroscopy can provide information on cell number, shape and chemical composition without sample labelling; however, it is difficult to extract the cell characteristics from the spectra. Besides, refractometry is a promising tool for label-free cell analysis, as the refractive index variation of the membrane can be correlated with the physiological or metabolic alteration of the cell, as in intracellular infection or malignant neoplasia [16,17,18,19].

In this context, the development of a microfluidic chip that integrates these four label-free detection methods, impedance, refractive index measurement, optical absorption and fluorescence, would provide a multifactorial analysis tool, suitable for a diversity of applications dealing with complex samples, ranging from environmental analysis to single cell analysis. In this paper, we describe the steps towards the implementation of a multifactorial analysis microfluidic device capable of such multifactorial analysis. Details are given on the design and fabrication of a hybrid microfluidic chip. To prove the feasibility of the developed multifactorial platform, we measured: (i) the impedance-based identification of bead size and the counting of erythrocytes; (ii) simultaneous analysis of the refractive index and absorption properties of solutions; and (iii) fluorescence-based bead counting.

## 2. Materials and Methods

### 2.1. Microfluidic Chip Fabrication and Characterization

The bottom part of the chip consists of an SU-8 layer patterned on a glass substrate. SU-8 2050 (Microchem, Newton, MA, USA) was spin coated (Karl Suss CT62, Munich, Germany) on the glass substrates (1 mm thick, Normax, Marinha Grande, Portugal) at 3000 rpm to form a ~53 μm-thick layer. Soft baking was done on a levelled hot plate for 1 min at 65 °C and then for 7 min at 95 °C. After cooling, the samples were ultraviolet (UV) exposed on a mask aligner (MA6 Suss MicroTec, Munich, Germany) with an exposure dose of 250 mJ/cm^2^ through an i-line filter (G180336 Suss MicroTec, Munich, Germany) and a photolithographic mask (chrome on soda lime glass, JD Photo-tools, Oldham, UK). The samples were post-baked during 1 min at 65 °C and subsequently for 6 min at 95 °C and then developed by submersing in propylene glycol methyl ether acetate (PGMEA, Microchem) during 5 min and 30 s with a magnetic agitation of 500 rpm, rinsing with isopropanol and drying gently with compressed nitrogen.

On the surface of the ~53-μm SU-8 layer, 2 pairs of electrodes were deposited by e-beam evaporation and patterned by photolithography and lift-off. The positive photoresist (AZ6612, AZ Electronic Materials, Wiesbaden, Germany) was spin coated at 3000 rpm for 10 s and then at 4000 rpm for 20 s. Soft bake was done for 1 min and 15 s at 110 °C. After cooling, the samples were exposed on the mask aligner for 3.5 s with an exposure power of 275 mW/cm^2^. The development was made in the AZ726 MIF developer (Microchemicals, Ulm, Germany) for about 30 s. Thereafter, a 20-nm titanium (22Ti) adhesion layer and a 100-nm gold layer were deposited by e-beam evaporation and then patterned by lift-off with acetone.

The top part of the chip was fabricated in polydimethylsiloxane (PDMS, Sylgard 184, Dow Corning, Midland, MI, USA) patterned by a two-layer SU-8 mold. For the first SU-8 layer, the SU-8 2025 was spin coated on silicon wafers at 4050 rpm to form a ~20-μm layer. Samples were then soft-baked (5 min at 95 °C), UV exposed through the i-line filter and a photomask (exposure dose: 145 mJ/cm^2^) and post-baked (1 min at 65 °C, then 5 min at 95 °C). For the second SU-8 layer, the SU-8 2025 was spin coated over the first SU-8 layer at 1650 rpm to form a ~53-μm coat. Subsequently, samples were soft-baked (1 min at 65 °C, then 5 min at 95 °C), UV exposed through the i-line filter and a photomask (exposure dose: 165 mJ/cm^2^). The pattern of the photomask was aligned with the pattern exposed in the first SU-8 layer. Subsequently, the samples were post-baked during 1 min at 65 °C and for 6 min at 95 °C and developed in PGMEA during 5 min and 30 s with a magnetic stirring of 500 rpm. To facilitate the PDMS de-molding, the mold was silanized with tridecafluoro-1,1,2,2-tetrahydrooctyl trichlorosilane (Microchem). 

PDMS was prepared by mixing a base and a curing agent in a 10:1 ratio of weight. Afterwards, the PDMS was degassed, poured over the SU-8 mold, degassed again and cured at 65 °C on a levelled oven for 2 h, and the PDMS was peeled off from the SU-8 mold.

For easier reproducibility of top part of the chips, a monolithic intermediate epoxy (ES562, Permabond, Pottstown, PA, USA) mold was produced to cast the PDMS, avoiding the degradation of the SU-8 related with delamination at the photoresist-substrate interface. The PDMS structures were placed on top of a Petri dish with the negative relief features up, upon which an epoxy resin was poured to form a ~2 mm-thick layer. After ~72 h of degassing in a desiccator to remove the bubbles, the epoxy glue was cured in an oven at 120 °C for ~40 min. Then, the cured epoxy was peeled from the PDMS and used as a master mold for PDMS soft lithography using the same procedure as described above. 

The alignment of the top and bottom parts of the chip was performed under a stereoscope magnifier Zoom 2000 (Leica, Solms, Germany) using a micrometer-driven aligner. A drop of methanol was added between both surfaces to facilitate the sliding; thus, after the alignment, the chip was rested for 10 min for methanol evaporation. The PDMS top part of the chip formed reversible sealing with the SU-8 bottom part by forming conformal contact though adhesion forces. The microfluidic devices were characterized by optical microscopy (Leitz Laborlux 12 ME ST, Wetziar, Germany), scanning confocal microscopy LSM 700 (Carl Zeiss AG, Oberkochen, Germany) and scanning electron microscopy FIB-SEM (Carl Zeiss AG).

### 2.2. Microfluidic Setup

**Figure 1:**
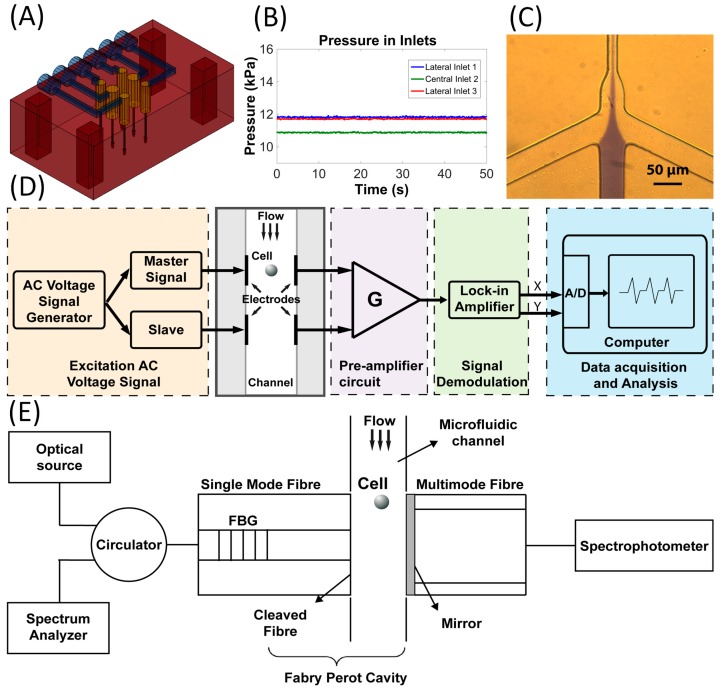
Characteristics of the developed microfluidic platform: (**A**) three-dimensional scheme of the poly(methyl methacrylate) (PMMA) acrylic chip holder; (**B**) the pressure in each inlet measured by the pressure monitoring system in an experimental assay; (**C**) hydrodynamic focusing situation in the central channel obtained using the previously-measured values; (**D**) schematic diagram of the complete microfluidic single cell impedance analysis system; and (**E**) scheme of the setup for the simultaneous measurement of the refractive index, absorption and fluorescence in the microfluidic chip.

To enable the movement of fluids and particles into the microchannels, a pneumatic pressure controller system was used. Acting on microliter liquid reservoirs located directly above a home-made poly(methyl methacrylate) (PMMA) chip holder (Figure 1A), a small volume of samples could be easily injected and guided into the microfluidic channels [20,21]. Moreover, an air pressure-driven system was built to control and measure the amount of applied pressure to each individual inlet/outlet of the microfluidic device (Figure 1B) and to easily obtain hydrodynamic focusing in the central channel (Figure 1C). The pressure supplier tubes were simultaneously connected to the microfluidic inlets and to the pressure transducers (Honeywell ASDX005PG Silicon Pressure Sensors, Morristown, NJ, USA) using PTFE tubing (Bohlender S1810-08, Grunsfeld, Germany) and silicon tubing connectors (Masterflex 96400-14, Vernon Hills, IL, USA) with auxiliary miniature connector accessories (Bohlender F710-01, Grunsfeld, Germany). Consequently, the pressure transducers were connected to an Arduino UNO R3 microcontroller (Arduino, Ivrea, Italy) monitored by the LabView (National Instruments, Austin, TX, USA) graphic user interface (GUI) for live recording of the data during the experimental assays. The pressure can be controlled from 0–0.2 bar (0–2.9 psi), and the movement of the fluids can be easily reversed, since the flux is a consequence of the pressure difference between inlets/outlets, without the risk of overpressure inside the channels in case of clogging. 

### 2.3. Impedance Analysis Setup

For the impedance measurement of cells, an alternating current (AC) discrete frequency system was used [22,23,24,25]. A schematic diagram of the setup can be seen in Figure 1D. An alternating electric field is established by applying an AC voltage (Thurlby Thandar Instruments TGA12100 Series, Huntingdon, UK) to the two pairs of planar electrodes positioned side by side. Two sinusoidal signals were generated with the same frequency and applied to the respective electrode, whereas one wave is the slave of the other (master), and its parameters, such as amplitude or phase, can be altered individually, but keeping the same frequency. This way, it can be possible to correct phase shifts between both waves. As particles pass through the two electric field regions, this consequently changes the current through each detection volume. The resultant signals from both electrode pairs are amplified and then subtracted by a home-made low-cost pre-amplifier circuit. The signal current amplification is made by a trans-impedance amplifier THS4601 (Texas Instruments, Madrid, Spain), which has a wide gain-bandwidth product (180 MHz), with a 100 kΩ feedback resistor. The subtraction of both signals is performed by an AD811 amplifier (Analog Devices, Munich, Germany) working as the voltage subtractor. Following that, another amplification stage was built. The output signal of the pre-amplifier circuit is inserted into a lock-in amplifier (EG&G Princeton Applied Research model 5202, Gaithersburg, MD, USA) where the de-modulation occurs, giving the real component (in-phase) and imaginary component (in quadrature) of the impedance. Afterwards, the lock-in amplifier is connected to an A/D converter (NI DAQ PCI6024E plus BNC2120, National Instruments, Austin, TX, USA) and the data captured and analyzed with software written in MATLAB (MathWorks, Natick, MA, USA) and LabVIEW.

### 2.4. Optical Setup

The simultaneous measurement of the refractive index and absorption/fluorescence was implemented by means of an interferometer and a spectrometer setup combined in the same chip by using fiber optic probes arranged as depicted in Figure 1E. From one side of the microfluidic channel (left side in Figure 1E), a standard telecom single mode fiber (SMF-28, Corning Optical Communications, Boulogne-Billancourt, France) was inserted, having a fiber Bragg grating (FBG) with a reflectivity of ~45% at 1550 nm inscribed in its core ~6 mm away from the tip. On the opposite side of the channel (right side in Figure 1E), a multimode fiber (50-μm core diameter), coated with a TiO_2_ mirror (obtained by e-beam evaporation) having ~50% reflectivity, was placed. With this arrangement, a low finesse Fabry–Pérot cavity was established between the FBG and the TiO_2_ mirror. An FS2200 laser scanning unit (FS2200, Fibre Sensing, Maia, Portugal) was used to retrieve the reflected interferometric pattern from which the refractive index of the flowing samples could be calculated. In particular, the sample refractive properties induce changes in the interference pattern (shift in the fringe position and changes in its contrast) from which its refractive index can be calculated.

At the same time, the partially mirrored fiber collects UV-Vis light into a CCD spectrometer (Ocean Optics, Dunedin, FL, USA) to perform absorption spectroscopy and fluorescence measurements. The setup was illuminated externally with a microscope tungsten lamp, and the chip was fixed in an imaging setup for characterization purposes. The control and data acquisition parameters were set by a dedicated LabVIEW program. 

### 2.5. Sample Preparation

Different types of samples were prepared to evaluate the multi-analysis capabilities of the chip. Two solutions of polystyrene beads were prepared: (i) containing 6-μm polystyrene beads (PeakFlow^TM^ Orange flow cytometry reference beads, 575 nm emission, Molecular Probes, Eugene, OR, USA) and (ii) containing 10-μm beads (Polystyrene microparticles PPs-10.0 G.kisher GbR, Steinfurt, Germany). In both solutions, a dilution of 1:100 in Dulbecco’s phosphate-buffered saline (PBS) 1× (pH = 7.2) (Gibco, Life technologies, Carlsbad, CA, USA) was used.

Red blood cells (RBCs) were collected from healthy lamb donors on a no-additive tube followed by a de-fibrination process. Blood was washed in a modified Vega y Martinez (mVyM) buffer by centrifugation at 450× *g* (gravity) for 10 min, until a clean supernatant was obtained. The white coat was removed, and the final pellet was resuspended 1:1 in mVyM buffer. A solution of 2% hematocrit was prepared on Medium 199 1× (Gibco, Life technologies). Tree aliquots were prepared in order to apply different conditions to each one: membrane permeabilization, membrane fixation and control (without treatment). Regarding the membrane permeabilization, 0.1% of Triton X-100 (Rohm and Hass Co., Philadelphia, PA, USA) in PBS was added to the erythrocyte suspension and incubated 5 min at room temperature. On the other hand, to permeabilize the erythrocytes’ membranes, 2% of glutaraldehyde (25% aqueous solution) (Sigma-Aldrich, St. Louis, MO, USA) in PBS was added and then incubated for 30 min at 4 °C. After the incubation period, both aliquots were centrifuged two times at 450× *g* for 5 min and thereafter washed and resuspended with PBS. For experimental tests, RBCs were diluted 1:3000 in PBS. For experimental assays in the microchannels, RBCs were diluted 1:3000 in PBS.

## 3. Results

### 3.1. Development of Microfluidic Chip

**Figure 2:**
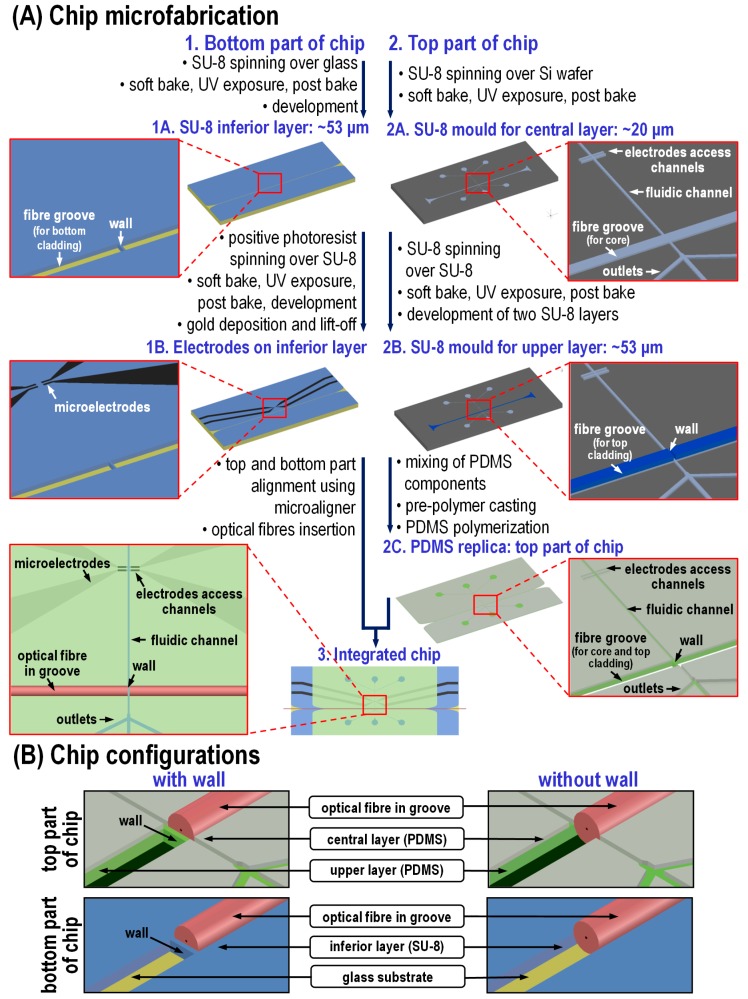
Construction details of the hybrid microfluidic chip: (**A**) microfabrication process (the bulk part of the PDMS is not shown so as not to darken the images) and (**B**) 3D schema of the optical detection area showing the configuration with the wall (**left**) and without the wall (**right**) in between the optical fibers (125 µm diameter) in the upper and inferior layers. The top part of the chip is placed upside-down on the bottom part of the chip forming a sandwich of a total fiber groove depth of 126 µm.

For refractometry and absorption analysis, optical fibers were used to transport light from the source to the microchannel and afterwards to the photodetector. Perpendicular to the fluidic microchannel, grooves for insertion of the fibers were placed for precise fiber alignment. The main challenge of the chip design was how to enable the microparticles or cell (here, erythrocytes, ~5-µm diameter) analysis using single-mode optical fibers. The commercially available single-mode optical fibers have an 8-µm core diameter; however, the core is embedded in a large cladding of 125 µm in diameter. To align the optical fibers, 126 µm × 126 µm grooves were patterned and placed in front of each other being separated by the fluidic channel (~20 µm wide). While analyzing uniform solutions is straight forward, when looking at cells or other microparticles, it is required that they should flow in a line in front of the optical fiber core; thus, the cell flow should be blocked below and above the fiber core. To do so, three-dimensional hydrodynamic focusing could be used; however, it requires the use of high flow rates that raises problems for the optical acquisition method towards refractive index measurements. Therefore, a multilayer three-dimensional chip has been designed to block the cells flow below and above the fiber core, setting the fluidic channel in the middle of the height of the fiber groove channel (Figure 2B).

To enable the analysis of ~5 µm large single erythrocytes, the cells should flow individually in the channel. To reduce the risk of channel clogging, the microchannel dimensions were set at 20 µm × 20 µm, and a lateral hydrodynamic focusing was used. We have demonstrated the impedance measurements of infected red blood cells in a channel of the same dimensions in our previous paper [26]. By setting the flow rates at ~500 and ~550 µm/s for the cell solution and the two sheath flows, respectively, we could reduce the width of the cell solution stream to 5.8 µm. Vertical hydrodynamic focusing was not used, as it would significantly increase the complexity of the microfabrication process. However, due to laminar flow, the majority of cells should flow mainly in the center of the channel.

The chip consists of three layers: (i) ~20 µm-thick central layer for fluid flow; (ii) ~53 µm-thick upper layer to block flow above the core; and (iii) ~53 µm-thick inferior layer to avoid the flow below the core (for details, see Figure 2B). The fluidic channel is patterned only in the ~20 µm-thick fluidic layer, while the fiber grooves are patterned in all of these layers, giving the aimed total groove depth of ~126 µm. 

**Figure 3:**
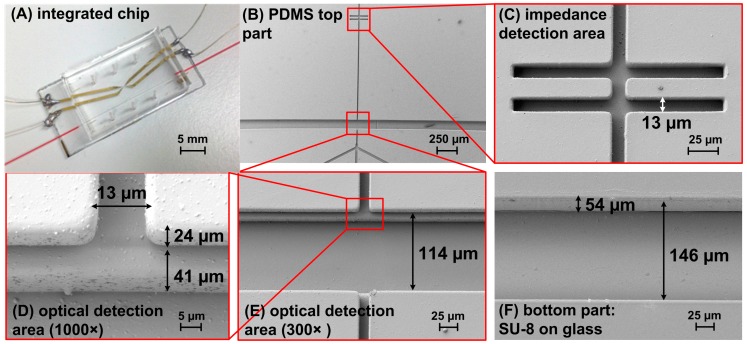
(**A**) Photography of a hybrid chip integrated with electrodes and optical fibers; (**B**–**F**) scanning electron micrographs of the components of the microfluidic chip showing the configuration without the wall in between the optical fibers: (**B**) top part of the chip fabricated in PDMS; (**C**) zoom-in showing the details of the impedance detection area; (**D**) optical detection area at magnification 1000× and (**E**) 300×; and (**F**) bottom part of the chip: SU-8 inferior layer on a glass substrate.

To enable the electrical characterization, microelectrodes were deposited by electron beam evaporation on the top of the ~53 µm-thick inferior layer, which after chip alignment is exactly on the bottom of the fluidic channel (Figure 2A and Figure 3A). This configuration assures that the cells flow in close proximity to the electrodes, increasing the sensitivity and contributing to solving the limitation described by Barat [9] where ~50% of cells were not analyzed electrically due to variations of the particle’s z-position in the large fluidic channel (80 µm deep).

To facilitate the microfabrication process, the chip was divided into bottom and top parts. The top part consists of the central and upper layers patterned in PDMS, a material of well-known advantages, such as low price, biocompatibility, high gas permeability and excellent optical properties: high optical transmittance down to 280 nm and low autofluorescence [27]. For the construction of the top part of the chip, the PDMS needs to be patterned by a mold that allows fabrication of: (i) high aspect ratio features to define grooves for fiber insertion and narrow channels for single cell or microparticle flow; (ii) features of smooth, vertical channel walls and side walls; and (iii) multilayer structures. These conditions can be satisfied by SU-8, an epoxy-based negative photoresist, which allows obtaining an aspect ratio above 20 and layering up to 650 μm by a single coating [28]. When patterning two SU-8 layers, they can be developed separately (the first layer before the spinning of the second SU-8 layer) or simultaneously at the end of the process (after post-bake of the second SU-8 layer). These two approaches have been tested towards achieving a high pattern visibility in the first SU-8 layer. The transparency of this layer is critical for the UV exposure of the second SU-8 layer: the features present in the first layer need to be aligned with the photomask pattern. It has been observed that the visibility of features present in the first SU-8 layer is much higher if it is not developed before the deposition of the second SU-8 layer. Otherwise, during spin coating, SU-8 fills up the features present in the first SU-8 layer, decreasing their contrast, and the pattern becomes almost invisible when observing under a mask aligner microscope; thus, the two SU-8 layers were developed simultaneously at the end of the process.

As after fabrication of a few PDMS replicas, the SU-8 mold suffers delamination at the photoresist-substrate interface, instead of directly producing the PDMS replicas from SU-8, an intermediate epoxy mold was applied due to its monolithic and stabile structure (i.e., without fragile material interfaces); see details elsewhere [29,30].

The bottom part of the chip consists of a glass substrate with a ~53 µm-thick SU-8 inferior layer with two pairs of electrodes deposited on it by electron beam evaporation. To reduce the chip cost, PDMS could be used as a structural material for the inferior layer, as electrodes can be patterned on its surface [31,32]. However, it has been observed that is much easier to pattern electrodes on the SU-8 surface due to its higher rigidity. Therefore, at this stage, SU-8 was used as a structural material for the inferior layer. The choice of SU-8 as the structural material for the bottom part of the chip has been guided by the conclusions from the literature review. Recently, SU-8 has been gaining increasing interest as a structural material for the fabrication of biological microdevices [33]. When compared to silicon wafers, SU-8 offers high mechanical strength and flexibility, chemical stability, high compatibility with polymeric packaging and encapsulation. As such, the patterning of electrodes on top of the flat SU-8 layer has already been achieved by several research groups [33,34,35,36,37]. In this work, the electrodes are also patterned on the flat part of the SU-8 layer. As such, the groove pattern defined in the 53 µm-thick SU-8 layer does not affect the lift-off process.

We have observed that the lift-off process on a 53 µm-thick SU-8 layer can be done in nearly the same way as on the flat glass substrate (we applied a gentle manual agitation and a brush). It is critical to assure a crack-free SU-8 surface, otherwise the cracks may anchor the metal layer, impeding the lift-off process. 

The adhesion of the electrodes to the SU-8 layer can be enhanced by the use of an adhesion-promoting metal (titanium, chromium or wolfram) as the interlayer between SU-8 and the actual electrode material [34]. In this work, we used a 20-nm titanium layer to promote the adhesion of the 100 nm-thick gold layer. We have observed no issues with the adhesion of the electrodes to the SU-8 layer.

As a substrate, 1 mm-thick glass was used due to its optical transparency, enabling imaging from the bottom of the chip by an inverted optical microscope. The overall chip dimension is 1.5 cm × 3.2 cm.

For refractometry measurements, the two fibers need to have their tips as close as possible. In this chip, the separation of the fiber tips is in the range of 20 μm, which ensures a good signal-to-noise ratio and accurate spectral measurement using a halogen lamp and a CCD integration time of hundreds of milliseconds [38]. Consequently, in the upper and inferior layers, the wall in between the fiber grooves is 126 µm long × 20 µm wide × 53 µm high (see the SEM image in Figure 3B,C). In the inferior layer, such a long and narrow SU-8 wall can easily break during fiber insertion, especially as the SU-8 has a poor adhesion to the glass [28]. To avoid this issue, a design geometry proposed by [39] has been adapted. They demonstrated that if the width of the sheathed sample is in the range of the particle diameter, the particles remain within the center line of the flow even in an expanding region. If so, a wall in between the optical fibers is not needed, as the cells would stay focused in the channel center when the 20 µm × 20 µm microchannel expands into the 20 µm × 126 µm microchannel in the optical detection area (see SEM image in Figure 3B). The two configurations, with and without the SU-8/PDMS wall in between the optical fibers, have been designed and fabricated.

The alignment of the top part of the chip with the bottom part was done using a stereoscope magnifier and a micrometer-driven aligner, taking usually a few minutes per chip. The addition of a methanol drop enables freely sliding the PDMS top part on the surface of the SU-8 bottom part. 

The produced chips were analyzed for their integrity and accuracy by optical microscopy, scanning electron microscopy and also scanning confocal microscopy. It has been observed that the features in the PDMS top part fabricated with the described process are narrower than in the SU-8 mould due to the PDMS shrinkage [40]. We obtained a channel width of 13 ± 2 μm, while the fiber groove width is 114 ± 4 μm, which were within the expected tolerance. Despite having obtained a fiber groove width narrower than the fiber diameter, the fibers can be easily inserted thanks to the PDMS elasticity. The thickness of the central layer is 24 ± 1 μm, whereas the thickness of the upper layer is 41 ± 2 μm, which is also in agreement with the expected values’ tolerance.

More critical for the fibers’ insertion are the dimensions of the fiber grooves in the SU-8 inferior layer, as SU-8 is rigid and may delaminate from the substrate during the insertion if the grooves are too tight. The grooves in the fabricated SU-8 inferior layer satisfy this requirement being 54 ± 6 μm high and 146 ± 10 μm wide. The total height of the grooves in the fabricated chip is 119 μm, which is suitable for the fibers’ insertion owing to the elasticity of the top part of the chip.

### 3.2. Electrical Analysis Results

For measuring the electrical impedance in the channel, the concept of “liquid electrodes” has been applied, in which the metal electrodes are positioned on the bottom of the perpendicular dead-end chambers apart from the central channel (see Figure 3C), as described by Demierre et al. [41]. This allows a good spatial resolution and increases the measurement range when compared to the classical designs of microelectrodes. Those narrow channels, which do the connection between the central channels and electrodes, are called access channels.

To perform the electrical measurements, a 5 V amplitude master AC voltage and an equal slave wave were applied by a waveform generator to the corresponding electrode pair. The system was calibrated injecting only a PBS medium solution into the microchannel, adjusting simultaneously the phase and amplitude between master and slave waves and the lock-in reference signal phase to obtain a continuous null resultant voltage signal at lock-in output. Due to the differential method scheme of the pre-amplifier circuit, when a particle passes through the electrical characterization zone, it induces changes in the two electric field regions, and a double-peak (one positive and one negative) signal shape is obtained. 

**Figure 4:**
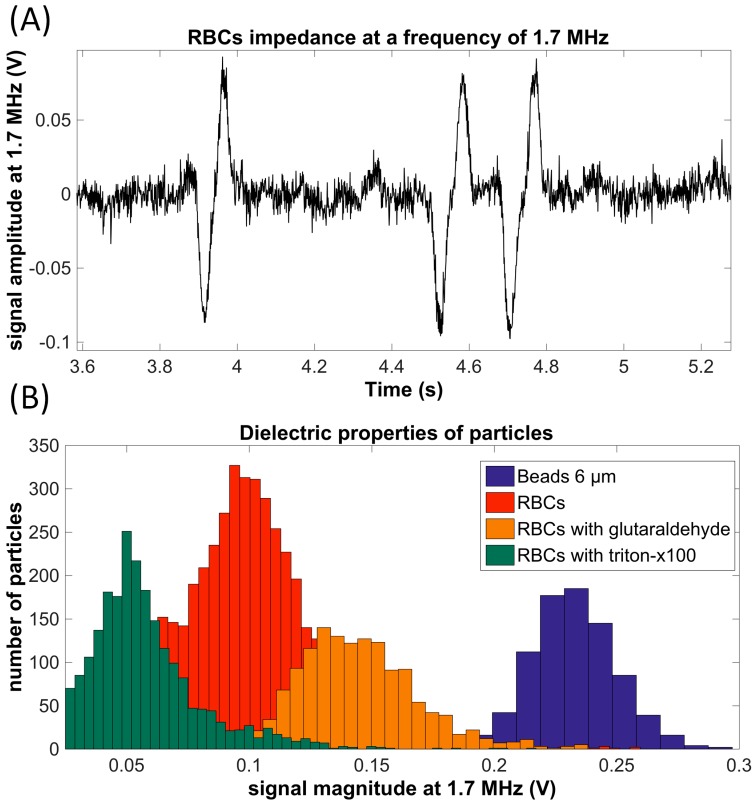
(**A**) Results obtained by passing red blood cells (RBCs) in the electrical characterization area at 1.7 MHz; (**B**) histogram of signal voltage magnitudes obtained by passages of different particles: 6-μm beads, RBCs, RBCs fixed with glutaraldehyde and RBCs fixed with triton.

After the system calibration with 6-μm and 10-μm microbeads at low frequencies, assays with hydrodynamically-focused erythrocytes (RBC) at room temperature were performed at 1.7 MHz (Figure 4A). Cells were injected into the chip with an approximate velocity of ~500 μm/s. An A/D conversion sample rate of 1000 samples per second was established.

Peaks were recorded and analyzed with a specific algorithm developed in MATLAB software. The results showed that the passing cells can be clearly discriminated by impedance measurements. Moreover, at this frequency range, particles could be distinguished by their volume and by membrane properties (Figure 4B), using this simple and low-cost electrical technique. 

### 3.3. Optical Analysis Results

**Figure 5:**
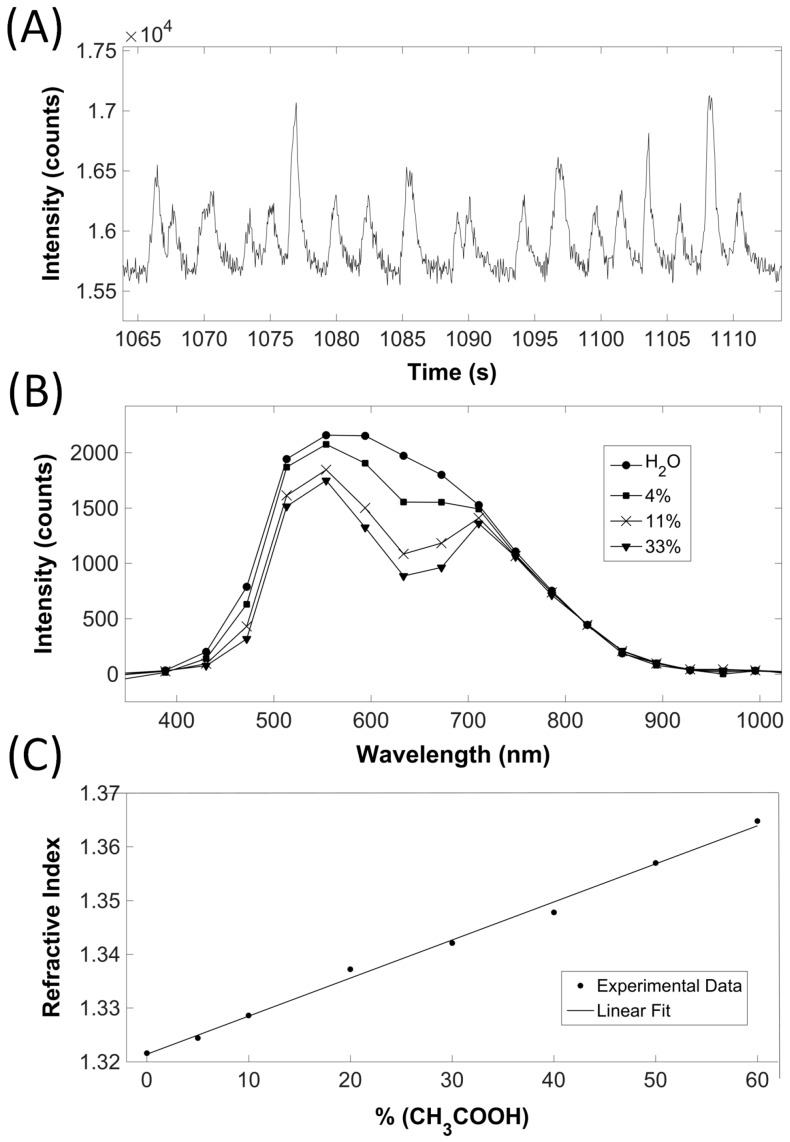
Optical characterization results obtained by the developed platform (see Figure 1E for the setup diagram): (**A**) counting of fluorescence beads (intensity corresponds to the sum of the detected charged-coupled device (CCD) counts over a 5-nm interval around the spectral emission peak at 582 nm); (**B**) absorption spectra of various malachite green-dyed solutions (each spectra corresponds to approximately 4000 data points with an associated uncertainty of ±2 counts); and (**C**) refractive index variation as a function of acetic acid concentration (each data point has an associated maximum uncertainty of 10^−4^ refractive index units).

To enable the simultaneous measurement of the refractive index, absorption and fluorescence in the microfluidic chip, we have previously tested various sensing configurations using different combinations of single-mode (SM) or multi-mode (MM) fibers with fiber Bragg gratings (FBG) and mirrors inserted into the microfluidic chip [38,40,41,42]. To obtain the sensing Fabry–Pérot (FP) cavity [16], one FBG inscribed in the SM fiber and one MM fiber with a tip covered by a partially reflecting mirror were assembled in the chip, as described in the experimental details (see also Figure 5). In this configuration, the SM fiber collects the interferometric signal in the near-infrared range (λ = 1550 nm), enabling the refractometric measurement, while the MM fiber collects the solution absorption/reflectance spectra and fluorescence signal in the visible range (400–800 nm) using the standard microscope illumination and collecting the signal with a standard CCD spectrometer. The reflectivity of the mirror and the FBG were set to 45% and 50%, respectively, to obtain both a good visibility of the interferometric fringes and a high signal-to-noise ratio in the absorption/fluorescence measurements. 

Figure 5 presents the optical analysis of the preliminary results obtained via the developed microfluidic platform. Using the MM detection fiber, fluorescent beads were successfully counted, analyzing the transmitted light that reached the CCD spectrophotometer (Figure 5A). The CCD was set to integrate the detected counts over a 5-nm interval around the spectral emission peak of the fluorophore at 582 nm and to display the corresponding intensity as a function of time. Each time a fluorescent bead passed by the fiber sensing area, an intensity peak was detected. In principle, weaker signals can be detected, corresponding to autofluorescence of cells, for instance. However, this will require larger integration times at the CCD, demanding much slower flow speeds or even immobilization of the cells at the sensing region.

To demonstrate the simultaneous analysis of the absorption and refractive index of solutions, we prepared a matrix of solutions with a gradual change of malachite green dye concentration and acetic acid concentration in deionized water. The acetic acid is expected to change the refractive index, while the dye determines the absorption peak. Using the MM detection fiber and the standard microscope lamp, we could observe changes in the absorption spectrum induced by various dye concentrations, which were in accordance with the Beer–Lambert law: at higher dye concentrations, a lower intensity of transmitted light in the range 600–700 nm, where the absorption peak for malachite green dye is located, could be observed (Figure 5B). Simultaneously to the acquisition of the solutions’ absorption spectra, the refractive index at 1550 nm was determined using the refractometric configuration (Figure 5C): as expected, in the range tested, the refractive index increases linearly with the acetic acid concentration. 

The observed behavior was well correlated with control measurements performed with a standard Abbe refractometer (2WAJ, PCE Instruments, UK), and a resolution of 10^−4^ was estimated and a calibration curve obtained. From these data and considering the samples having no acetic acid, it could be observed that the increasing concertation of the dye, alone, was responsible for modifications in the measured refractive index (a 0.0429 change was recorded for a concentration increasing from 0.1%–27.0%). With this configuration, the possibility of the simultaneous refractive index and absorption measurements of complex liquid solutions was demonstrated. However, although the recorded refractive index resolutions were enough for single cell measurements, the analysis of the refractive index of heterogeneous solutions containing beads or cells in the flow, using the present configuration, requires stopping of the targets in front of the optical fiber sensing region, for a specific period of time. This depends on the characteristics of the acquisition unit (here: FS2200 Braggmeter, Fiber Sensing, Maia, Portugal), where at least one second is necessary. Therefore, to allow measurements of the characteristics of beads or cells, a higher processing speed of the optical instrumentation and data collection is needed. Optical or electrostatic trapping of the cells in the measurement region is possible, but not suitable for a large number of cells. Alternatively, much faster and accurate measurements of the refractive index can be implemented using the same setup, equipped with an interferometric interrogation scheme. Previous experiments demonstrated that systems such as white light interferometers enable the readout of interferometric signals with at least an order of magnitude improvement in the resolution and speed of measurement, as compared with spectral methods [43].

## 4. Conclusions

A novel PDMS/SU-8-based microfluidic platform was designed and fabricated for the characterization of complex samples enabling simultaneous measurements of impedance, refractive index, optical absorption and fluorescence. The integration of electrodes and optical fibers in a miniaturized device allowed analysis using a few microliters of sample. The system was characterized by validating each of the analytical techniques with different types of samples. 

Fluorescence detection was demonstrated by counting of fluorescent beads, which indicates the system capability for fluorescence-based cytometry. In principle, higher sensitivity measurements are possible by increasing the CCD integration time, enabling, for instance, the detection of cells’ autofluorescence. Such an approach, however, demands stopping the cell at the fiber sensing region. While this is limiting considering standard flow cytometers, it can be of great interest in the long-term monitoring of single cells. In addition, the fluorescence detection ability can be very useful to incorporate fluorescence-based analytical methods, using indicator dyes, for the detection of specific analytes in environmental samples or in the cell microenvironment.

The impedance analysis feature of the hybrid chip was also validated: red blood cells were identified and counted by impedance analysis at 1.7 MHz, which could enable sizing and membrane properties’ discrimination.

With the aim to provide multifactorial analysis, simultaneous measurement of the refractive index and absorption spectroscopy of dyed acetic acid solutions were also demonstrated. A calibration was performed against a standard Abbe refractometer, and the simultaneous measurements allowed determining the influence of the dye concentration on the measured refractive index. Such capabilities are very promising, as the measurement of the refractive index of the bulk solution can be of great interest either for cell analysis or for a diversity of other analytical applications dealing with complex solutions. For instance, in certain experimental situations, the acetic acid, or other chemical species, can be a result of a metabolic reaction of the cell under analysis, or can indicate the presence of a certain chemical compound in an environmental analysis (which can benefit also from many available colorimetric indicators). Regarding the refractive index measurements of the cell itself, it requires immobilization of the particles in front of the SM fiber core for at least 1 s, which is not suitable for samples with a large number of particles/cells. As such, a higher processing speed of the optical instrumentation and data collection is needed to enable determination of the cells’ refractive index. However, alternative interferometric interrogation methods are available, which can enable faster higher resolution measurement. 

Overall, the obtained preliminary results allowed establishing the developed platform as suitable for the analysis of complex samples using multiple electrical and three optical characterization techniques. Several technological challenges arising from the integration of widely different sensing schemes were identified, and hints were given for further upgrades. Further optimization of the hybrid chip platform should enable its application in a diversity of applications ranging from environmental analysis to single cell diagnostic.
